# Polymorphisms of heat shock protein 70 genes (HSPA1A, HSPA1B and HSPA1L) and susceptibility of noise-induced hearing loss in a Chinese population: A case-control study

**DOI:** 10.1371/journal.pone.0171722

**Published:** 2017-02-09

**Authors:** Yanhong Li, Shanfa Yu, Guizhen Gu, Guoshun Chen, Yuxin Zheng, Jie Jiao, Wenhui Zhou, Hui Wu, Zengrui Zhang, Huanling Zhang, Lihua He, Qiuyue Yang, Xiangrong Xu

**Affiliations:** 1 Henan Provincial Institute for Occupational Health, Zhengzhou, People’s Republic of China; 2 Wugang Institute for Occupational Health, Wugang, People’s Republic of China; 3 National Institute of Occupational Health and Poison Control, Chinese Center for Disease Control and Prevention, Beijing, People’s Republic of China; 4 Department of Occupational Health and Environmental Health, School of Public Health, Peking University, Beijing, People’s Republic of China; Duke Cancer Institute, UNITED STATES

## Abstract

Noise-induced hearing loss (NIHL) is the second-most frequent form of sensorineural hearing loss. When exposed to the same noise, some workers develop NIHL while others do not, suggesting that NIHL may be associated with genetic factors. To explore the relationship between single nucleotide polymorphisms (SNPs) in heat shock protein 70 (HSP70) genes (HSPA1A, HSPA1B and HSPA1L) and susceptibility to NIHL in Han Chinese workers exposed to noise, a case-control association study was carried out with 286 hearing loss cases and 286 matched with gender, age, type of work, and exposure time, drawn from a population of 3790 noise-exposed workers. Four SNPs were selected and genotyped. Subsequently, the effects of the alleles and genotypes of the three HSP70 genes (HSPA1A, HSPA1B and HSPA1L) on NIHL were analyzed by using a conditional logistic regression. A generalized multiple dimensionality reduction (GMDR) was applied to further detect an interaction between the four SNPs. Compared with the combined genotypes CC/TC, carriers of the TT genotype of rs2763979 appeared to show greater susceptibility to NIHL (*P* = 0.042, adjusted *OR* = 1.731, *95% CI* 1.021–2.935). A significant interaction between rs2763979 and CNE was found (*P* = 0.029), and a significant association was found between TT of s2763979 and NIHL (*P* = 0.024, adjusted *OR* = 5.694, *95%CI* 1.256-25.817) in the 96 dB (A)≤CNE<101 dB (A) group. The results suggest that the rs2763979 locus of the HSP70 genes may be associated with susceptibility to NIHL in Chinese individuals, and other HSP70 genes may also be susceptibility genes for NIHL, but the results must be further replicated in additional independent sample sets.

## Introduction

Noise-induced hearing loss (NIHL) is caused by regular exposure to continuous noise or exposure to a single acoustic overstimulation. Since the industrial revolution, an increasing number of people are exposed on a daily basis to harmful levels of noise in their work environment. As a result, NIHL is the second-most common form of sensorineural hearing loss after presbycusis [1]. Thirty-five million people are at risk for developing NIHL in Europe alone [2]. Ten million people in the United States have noise-related hearing loss, and the number of workers in China with NIHL has increased 77.8% in the past three years (2010–2012) [3].

NIHL is a complex disease caused by a combination of environmental and genetic factors. Noise >85 dB is harmful and causes both mechanical and metabolic damage [4]. Workers who are occupationally exposed to high levels of noise possess high rates of NIHL [5]. In addition, individual factors such as smoking, high blood pressure and cholesterol levels may influence the susceptibility to noise [5,6,7,8,9].

Not all workers exposed to the same levels and duration of noise will develop NIHL, and the degree of hearing loss varies widely, indicating that NIHL may be associated with genetic factors. Association studies have identified GSTM1, GSTT1, SOD1, SOD2, KCNE1, PON2, CAT, CDH23 and HSP70 as putative NIHL susceptibility genes [8,10,11,12,13,14,15,16,17]. However, some studies involving other populations found no significant association between NIHL and GSTM1, GSTT1 or SOD2 [18,19]. Heat-shock proteins (HSPs) that perform housekeeping and quality control functions in the cell [20] are expressed ubiquitously in cells under both physiological and pathological circumstances. The human HSP70 family is composed of three genes: HSPA1A, HSPA1B and HSPA1L [21]. All three are expressed in the cochlea after acoustic overstimulation [22].

In this study, we investigated whether we could replicate the associations observed between the single nucleotide polymorphisms (SNPs) in the HSP70 genes (HSPA1A, HSPA1B and HSPA1L) and susceptibility to NIHL in Han Chinese workers exposed to noise.

## Materials and methods

### Samples

A total of 3790 Han workers exposed to continuous and steady-state occupational noise in a steel factory in the Henan province of China participated in this study. Cases and controls were selected as follows. First, according to the criterion that the average binaural hearing threshold level (HTL) to high frequency (3000 Hz, 4000 Hz and 6000 Hz) is greater than or equal to 40 dB or the average of single hearing threshold level to linguistic frequency (500 Hz, 1000 Hz and 2000 Hz) is greater than or equal to 26 dB, 479 cases were included from the 3790 subjects, and 958 controls who were frequency-matched by gender, age, type of work, and exposure time were also included from the remaining 3311 subjects. Second, 6 cases with no blood samples and their 12 controls were excluded. Third, an inclusion criterion for this study was an exposure to occupational noise higher than 80 dB(A) for more than 3 years. Subjects with a history of explosive noise exposure, skull trauma, contagious diseases (mumps, measles or rubella), middle ear disease, Meniere's disease, treatment with an ototoxic drug, perforated eardrum, or with a family history of hearing loss and subjects of non-noise-induced hearing loss were excluded. Additionally, controls with the average of binaural HTL of high frequency ≥35 dB or unilateral HL of any linguistic frequency >25 dB were excluded, including 6 cases and 18 controls whose noise exposure time was less than 3 years, 9 cases and 11 controls with a history of exposure to explosive noise, 5 cases and 6 controls with a history of skull trauma, 68 cases and 132 controls with a history of mumps, 9 cases and 17 controls with a history of measles, 24 cases and 67 controls with a history of middle ear disease, 3 cases with a history of Meniere's disease, 1 control with a history of treatment with an ototoxic drug, 8 cases and 4 controls with a history of a perforated eardrum, 39 cases of non-noise-induced hearing loss that were excluded by an expert in the diagnosis of noise deafness according to the standards provided in the ‘Diagnostic Criteria of Occupational Noise-induced Deafness’ (Chinese Occupational Health Standard, GBZ49-2007), 4 cases and 2 controls with a family history of hearing loss, 43 controls with an average binaural HTL of high frequency ≥35 dB, and 33 controls with a unilateral HTL of any linguistic frequency >25 dB. Furthermore, 298 cases were further matched according to the same criterion above, and 12 cases that could not be well-matched were also excluded. Finally, 1:1 pairing case-control method was adopted with 286 cases and 286 controls. The study was approved by the Ethics Committee of Henan Institute of Occupational Medicine. Participants provided their written informed consent to participate in this study.

### Physical examination and epidemiological survey

Using a mercury sphygmomanometer, trained physicians performed a physical examination on each subject to measure systolic and diastolic blood pressure levels by following a standard protocol. Through face-to-face interviews, trained investigators collected by questionnaire the individual demographic features of each subject, including age, gender, smoking habits, drinking habits, history of disease, history of tympanic membrane perforation or skull trauma, family history of hearing loss, history of drug use, type of employment, duration of noise exposures during working hours, and noise exposure in previous workplaces and in the army.

### Audiological status assessment and environmental noise measurement

According to the standards provided in the ‘Diagnostic Criteria of Occupational Noise-induced Deafness’ (Chinese Occupational Health Standard, GBZ49-2007), an As216 audiometer (Interacoustics AS Company, Danish) was applied to measure the air and bone conduction threshold audiometry for both ears at 0.5, 1, 2, 3, 4 and 6 kHz. All audiometry was performed by trained occupational health physicians in a quiet cabinet that guaranteed a background noise value <25 dB(A). Before undergoing audiometry, all subjects were required to have been removed from an occupational noise environment for >12 hours. The results were adjusted for gender and age according to the Occupational Health Standard of the People’s Republic of China: Acoustics-statistical distribution of hearing thresholds as a function of age (GB/T7582). Apart from audiometry, an otology morphological examination and otoscopy were requested, including assessment for bilateral auricle malformation, external auditory canal malformation and stenosis, tympanic membrane perforation, and adhesion or calcification, among others.

The intensity of personal noise exposure was assessed from 8 a.m. to 4 p.m. during the work shift using Noise Dose Meters and a QC-10 sound level calibrator (NoisePro series, Quest Technologies, American). The measurement methods and equivalent continuous dB(A)-weighted sound pressure levels (LEX, 8 h) conformed to the Occupational Health Standard of the People’s Republic of China: Measurement of Noise in the Workplace (GBZ/T 189.8–2007). To determine the total noise exposure level, the cumulative noise exposure (CNE) was calculated according to monitoring data on A-weighted sound pressure level and employment time as follows [23]:

$$CNE=10Lg\left[\frac{1}{Tref}\sum_{i=1}^{n}\left(Ti\times 10^{LAeq\frac{8hi}{10}}\right)\right]$$

where Ti is the duration of the work history i (years); L_Aeq,8hi_ is the equivalent continuous dB(A)-weighted sound pressure levels in decibels normalized to an 8-hr working day; and T_ref_ = 1 year.

### Blood collection and DNA isolation and determination of purity

Peripheral blood (2 ml) was collected from each subject in a 2-ml EDTA anticoagulant tube by trained physicians and stored in a -80°C refrigerator. Genomic DNA was obtained from peripheral blood samples using a 2 ml DNA Extraction Kit (Shanghai Lifefeng Biotech Co., Ltd, Shanghai, China) according to the manufacturer’s protocol. A NanoPhotometer P360 (Shanghai Boyibio Biotech Co., Ltd., Shanghai, China) was used to determine the concentration and purity of the resultant genomic DNA. Only the DNA samples with an A260/A280 ratio between 1.8 and 2.0 and a concentration >50 ng/μl were used subsequently for genotyping.

### SNP selection and genotyping

SNPs were selected based on the HapMap database (http://hapmap.ncbi.nlm.nih.gov/) and dbSNP (http://www.ncbi.nlm.nih.gov/snp/). Inclusion criteria were as follows: SNPs located in the entire region of the HSP70 genes (HSPA1A, HSPA1B and HSPA1L) with a minor allele frequency (MAF) >0.10 in Han Chinese, covering 2 kb upstream of the transcription start site and all exons and introns. Then, a linkage disequilibrium (LD) method was applied to select tagged SNPs with a pairwise r^2^ >0.80 as candidate SNPs. Finally, rs1043618 in HSPA1A, rs2763979 in HSPA1B, and rs2075800 and rs2227956 in HSPA1L were selected for analysis.

A SNPscan^™^ multiplex SNP genotyping kit (Genesky Biopharm Technology Co., Ltd., Shanghai, China) was employed to detect the genotypes of these four loci among the 572 samples. SNPscan^™^ provides an accurate and high-throughput method for genotyping 48-192 SNPs simultaneously that involves double ligation and multiplex fluorescence PCR. Briefly, a multiplex fluorescence PCR reaction was performed with a mixture, including DNA, PCR Master Mix (2×), Primer Mix I or II, the ligation product and ddH2O. The procedure consisted of the following steps: an initial denaturation of 2 minutes at 95°C; 9 cycles of 20 seconds denaturation at 94°C, annealing at 62°C (decrease by 0.5°C per cycle) for 40 seconds, and an extension for 1.5 minutes at 72°C; followed by 25 cycles of 20 seconds of denaturation at 94°C, annealing at 57°C for 40 seconds, and extension at 72°C for 1.5 minutes. A final extension was performed at 68°C for 60 minutes. After a 5 minute denaturation at 95°C, PCR products were sequenced by an ABI3730XL DNA analyzer, and the results were analyzed by GeneMapper 4.1 software (Applied Biosystems, USA). Genotyping was performed in a blinded fashion without knowing any information about the subject.

### Statistical analysis

Hardy—Weinberg equilibrium (HWE) tests were performed using Pearson’s χ^2^ for each SNP. Continuous data were analyzed by a paired samples t-test. Four genetic models were used, including an additive-inheritance model, a dominant-inheritance model, a recessive-inheritance model and a co-dominant-inheritance model. A conditional logistic regression was used to test associations between genetic frequency and disease status, with adjustments for the possible confounding variables, to obtain adjusted odds ratios (ORs) for risk of NIHL and their 95% confidence intervals (CIs). Interaction effects between genotypes and individual/environmental factors were tested by inclusion of an interaction term (genotype × individual/environmental factors) in the logistic model. In cases where the interaction term genotype × individual/environmental factor was significant, the main effect of the genotype was analyzed for each separate stratum. The associations of genotypes with NIHL were stratified by CNE, all noise exposed workers were divided into three categories (CNE<96dB[A], 96dB[A]≤CNE<101dB[A] and CNE≥101 dB[A]). GMDR software (version 0.9, http://sourceforge.net/projects/gmdr/) was applied for further detecting the best model associated with NIHL. All statistical analyses were two-sided and were performed by using SPSS 20.0 (IBM SPSS Statistics 20 for Windows, Chicago, IL, USA http://www.stat.washington.edu/stephens/home.html), and the significance level was set at 0.05.

## Results

### Analysis of the equilibrium factors in the case and control groups

The comparison of the equilibrium factors in the case and control groups is presented in Table 1. There was no significant difference between the case and control subjects in age, duration of noise exposure, CNE or gender (*P* > 0.05). The case and control subjects were considered to be well-matched. There was no significant difference between the case and control subjects in the rate of hypertension or drinking status (*P* = 0.933 and 0.474, respectively). There was a significant difference in the distribution of smoking status between the case and control subjects (*P* < 0.001). Variables (blood pressure, drinking status and smoking status) were further adjusted in the conditional logistic regression.

**Table 1:** Comparison of the equilibrium factors in the case and control groups.

Variables	Case	Control	*P*
Age(yr), mean ±SD	40.5±8.1	39.8±8.1	0.298
Tenure(yr), mean ±SD	18.9±9.1	18.3±8.8	0.382
CNE, mean ±SD	98.3±4.7	98.3±4.6	0.999
Gender, n (%)			
male	274(95.8)	274(95.8)	1.000
female	12(4.2)	12(4.2)	
Hypertension, n (%)			
yes	121(42.3)	122(42.7)	0.933
no	165(57.7)	164(57.3)	
Smoking, n (%)			
yes	192(67.1)	141(49.3)	**<0.001**
no	94(32.9)	145(50.7)	
Drinking, n (%)			
yes	198(69.2)	190(66.4)	0.474
no	88(30.8)	96(33.6)	

### Evaluation of the genotype effect on the disease status

Basic information about the four SNPs in the three HSP70 genes is shown in Table 2. All four SNPs were in HWE (*P* >0.05), indicating that subjects were in equilibrium from generation to generation. As shown in Table 3, two SNPs showed significant differences in genotype frequency between NIHL case and control groups. For rs2763979, compared with the CC genotype, the TT genotype had a significantly decreased effect on NIHL risk (*P* = 0.036, adjusted *OR* = 1.859, *95% CI* 1.042–3.315) and compared with the combined genotypes CC/TC, carriers of the TT genotype appeared to be more susceptible to NIHL (*P* = 0.042, adjusted *OR* = 1.731, *95% CI* 1.021–2.935). For rs2227956, compared with the combined genotypes AA/GA, carriers of the GG genotype appeared to be more susceptible to NIHL in recessive effect (*P* = 0.039, adjusted *OR* = 5.502, *95% CI* 1.093-27.694). For rs1043618 and rs2075800, there were no significant differences in the distributions of variant models and alleles between the case and control subjects.

**Table 2:** Characteristics of single nucleotide polymorphisms of genetic analysis.

Genes	SNPs	Nucleotide change	Location	MAF(HapMap-CHB)	*P*(H-W)^a^
HSPA1A	rs1043618	C/G	5'UTR	0.311	0.907
HSPA1B	rs2763979	C/T	5'_flanking	0.333	0.263
HSPA1L	rs2075800	C/T	exon2	0.314	0.759
	rs2227956	A/G	exon2	0.204	0.091

^a^ HWE tests were performed using Pearson’s χ2 for each SNP among the control subjects.

**Table 3:** Single SNP analysis of the association of HSP70 gene polymorphisms with the risk of NIHL.

SNPs	Genotypes	Case(n = 286)		Control(n = 286)		*P*	Adjusted OR (95% CI)*
		N	%	N	%		
rs1043618	GG	124	43.4	130	45.5		1.000
	GC	117	40.9	125	43.7	0.883	0.973(0.677–1.399)
	CC	45	15.7	31	10.8	0.207	1.432(0.820–2.500)
	GC/CC	162	56.6	156	54.5	0.738	1.060(0.754–1.491)
	GG/GC	241	84.3	255	89.2		1.000
	CC	45	15.7	31	10.8	0.169	1.450(0.853–2.469)
	Additive					0.366	1.122(0.874–1.440)
	G allele	365	63.8	385	67.3		1.000
	C allele	207	36.2	187	32.7	0.356	1.100(0.898–1.348)
rs2763979	CC	104	36.4	116	40.6		1.000
	TC	133	46.5	139	48.6	0.556	1.124(0.763–1.656)
	TT	49	17.1	31	10.8	**0.036**	1.859(1.042–3.315)
	TC/TT	182	63.6	170	59.4	0.253	1.243(0.856–1.803)
	CC/TC	237	82.9	255	89.2		1.000
	TT	49	17.1	31	10.8	**0.042**	1.731(1.021–2.935)
	Additive					0.059	1.297(0.990–1.699)
	C allele	341	59.6	371	64.9		1.000
	T allele	231	40.4	201	35.9	0.053	1.271(0.997–1.621)
rs2075800	CC	128	44.8	112	39.2		1.000
	TC	128	44.8	132	46.2	0.323	0.832(0.579–1.197)
	TT	30	10.5	42	14.7	0.056	0.576(0.328–1.013)
	TC/TT	158	55.2	174	60.8	0.156	0.777(0.548–1.101)
	CC/TC	256	85.9	244	85.3		1.000
	TT	30	10.5	42	14.7	0.196	0.642(0.281–1.081)
	Additive					0.061	0.780(0.602–1.011)
	C allele	384	67.1	356	62.2		1.000
	T allele	188	32.9	216	37.8	0.129	0.854(0.697–1.047)
rs2227956	AA	204	73.9	201	72.8		1.000
	GA	64	23.2	73	26.4	0.455	0.849(0.552–1.305)
	GG	8	2.9	2	0.7	0.058	4.784(0.949–24.113)
	GA/GG	72	26.1	75	27.2	0.695	0.919(0.604–1.400)
	AA/GA	268	97.1	274	99.3		1.000
	GG	8	2.9	2	0.7	**0.044**	5.204(1.047–25.866)
	Additive					0.770	1.059(0.722–1.553)
	A allele	472	85.5	475	86.1		1.000
	G allele	80	14.5	77	13.9	0.824	1.033(0.779–1.368)

* Adjusted for blood pressure, drinking status and smoking status

### Interaction and stratification analysis of HSP70 genes by individual/environmental factors

The P-values for the interaction between the genetic and individual/environmental factors analyzed for the SNPs are shown in S1 Table. A significant interaction between rs2763979 and CNE was identified (P = 0.029).

In order to reveal the interactions between SNPs and noise exposure, we performed stratified analysis by CNE which were shown in Table 4 (presented only for the SNPs with significant results). For rs2763979, comparing with CC/TC genotypes, the TT genotype increased the risk of NIHL (*P* = 0.024, adjusted *OR* = 5.694, *95%CI* 1.256–25.817) in the 96 dB(A)≤CNE<101 dB(A) group.

**Table 4:** Association between rs2763979 and NIHL stratified by CNE.

CNE	Genotypes	Case(n = 286)		Control(n = 286)		*P*	Adjusted OR (95% CI)*
		N	%	N	%		
<96	CC/TC	85	85.9	90	89.1	0.103	1.000
	TT	14	14.1	11	10.9		2.784(0.813–9.542)
96–101	CC/TC	77	82.8	89	92.7	0.024	1.000
	TT	16	17.2	7	7.3		5.694(1.256–25.817)
≥101	CC/TC	75	79.8	76	85.4	0.351	1.000
	TT	19	20.2	13	14.6		1.611(0.592–4.390)

* Adjusted for smoking status, drinking status and hypertension status

### Evaluation of the interaction effect between SNPs

To test the interactions between these SNPs, GMDR were performed. As shown in Table 5, a sign test indicated that there was no best model after adjustment for covariates, including blood pressure, drinking status and smoking status (*P* >0.05).

**Table 5:** The best combination models identified by the GMDR.

Locus No.	Best Combination	Cross-validation consistency	Testing balanced accuracy	*P*(sign test)*
1	rs2075800	5/10	0.4925	0.8281
2	rs2075800,rs2227956	10/10	0.5769	0.0547
3	rs2763979, rs2075800, rs2227956	8/10	0.5409	0.3770
4	rs1043618, rs2763979, rs2075800,rs2227956	10/10	0.5182	0.3770

* Adjusted for blood pressure, drinking status and smoking status

## Discussion

Four SNPs located in three genes of the HSP70 family were analyzed in a Chinese sample group in this study. Compared with the combined genotypes CC/TC, carriers of the TT genotype of rs2763979 appeared to be more susceptible to NIHL, and the rs2227956 locus of the HSP70 genes may be associated with susceptibility to NIHL. These results can be obtained by analyzing S1 Data. Rs2763979 is located in the HSPA1B 5' flanking region that is non-coding but plays a role in the control of gene expression. The rs1043618 and rs2075800 loci of the HSP70 genes were not associated with NIHL. Polymorphisms in HSP70 genes had previously been investigated in different populations [17, 24, 25], and significant associations with NIHL were found although the loci were not all the same. This indicates that the association between HSP70 genes and NIHL has been replicated in independent populations. Replication in independent sample sets is crucial to confirm the susceptibility genes for complex diseases.

Yang et al (2006) did not detect a statistically significant difference in the genotype and allele distributions of rs1043618, rs1061581, or rs2227956 between the NIHL group and the normal group. However, the investigators only found significant associations between NIHL and two haplotypes, GGT and GGC. This result is consistent with the distributions of rs1043618 and rs2227956 between the case and control groups in this study, although the inclusion criteria were different and subjects were from different industries. The GC genotype of rs1043618 was associated with NIHL susceptibility in a Taiwanese population, but no significant association was found for rs2763979 and rs2075800 [24]. For rs2075800, this result was similar to that of Chang. However, for rs1043618 and rs2763979, this result was different from Chang. The rs2227956 locus of the HSP70 genes showed a significant association with NIHL in both Swedish and Polish populations, and another two loci from the HSP70 genes (rs1043618 and rs1061581) were significant in the Swedish sample set [17]. These differences may be due to several factors as follows. First, there are substantial ethnic differences between the sample sets. MAF of SNPs is well-known to differ between populations of different ancestry. For rs1043618, the MAF for the HapMap Chinese population is comparable with the MAF for the HapMap European population (0.311 and 0.342, respectively; www.hapmap.org). For rs2227956, C is the minor allele with a MAF of 0.189 in the Asian population and 0.292 in the European population as reported on the NCBI website (http://www.ncbi.nlm.nih.gov/). The results can be regarded as a replication if the association between a disease, and the same gene was similar considering that different SNPs are associated in different populations [26]. Thus, this study can be regarded as a replication, which confirmed an association of HSP70 genes with NIHL. Second, these sample sets were gathered from different kinds of industries. The subjects of Chang’s study were noise-exposed workers from six similar petrochemical factories (synthetic resin, rubber and plastic factories). Polish noise-exposed workers came from different industries, including a coal mine, an electric power station, a dockyard, a glass bottle factory and a lacquer and paint factory. Swedish noise-exposed workers came from two paper pulp mills and one steel factory in the county of Värmland in the middle-west part of Sweden, and Chinese noise-induced workers came from a steel factory. Third, different research methods were adopted.Chang et al (2011) adopted a comparative research method between noise-susceptible individuals and non-susceptible subjects. Konings et al (2009) adopted a comparative research method between sensitive subjects and resistant subjects, while this study adopted a case-control study design. Last but not least, the size of the sample set was different.Our sample size in this study included 286 cases and 286controls in total and was larger than the Polish sample set that included 119 resistant subjects and 119 sensitive subjects, and the Swedish sample set consisted of 98 noise-susceptible and 108 noise-resistant subjects.

NIHL is well known as a multi-factorial disease and noise is the most frequent cause of NIHL. So the P-values for the interaction between genetic and individual/environmental factors were analyzed and a stratified association analysis was conducted between SNPs and NIHL by CNE. A significant interaction between rs2763979 and CNE was identified. After grouping the participants by CNE, the genotype effect for each separate stratum of CNE was calculated. A significant association was found between TT and NIHL in the 96 dB(A)≤CNE<101 dB(A) group. These results indicate that polymorphisms in rs2763979 in the HSP70 genes have a larger effect when laborers are exposed to mid-level CNE.

In addition to individual SNPs, we carried out a GMDR analysis to assess the impact of interactions between the four SNPs. The main effect of factors can be found by logistic regression, but interactions between factors cannot be evaluated inadequately due to the “curse of dimensionality.” GMDR has reasonable power for detecting interactions and permits adjustment for discrete and quantitative covariates. The Sign test indicated that there was no best model after adjustment for covariates, including blood pressure, drinking status and smoking status (*P* >0.05).

Previous studies have investigated the relationship between the SNPs within the HSP70 genes and susceptibility to NIHL mostly by comparing the difference between vulnerable and tolerance groups, while a case-control study was less often used. Case and control subjects were strictly frequency-matched by gender, age, type of work, and exposure time in this study. However, other potential factors, such as smoking status, drinking status and hypertension status, were not frequency-matched between the case and control groups. Considering the difficulty of pair matching, other potential confounding factors were controlled by the multivariate logistic regression analysis. None of the experimental (DNA extraction and genotyping) operation personnel and participants knew the patient’s disease status, therefore, the results were relatively objective.

Previous studies have found that other diseases besides NIHL, such as autoimmune diseases [27], acute high altitude illness [28], ischemic stroke [29] and postoperative atrial fibrillation [30], were associated with HSP70 genes, which indicates that HSP70 genes may act in many stress-related diseases. However, the underlying physiological mechanisms how the genotypes of HSP70 genes do (or do not) impose effects on NIHL is unclear. Thus, performing more in vivo and in vitro research on the association between polymorphisms of HSP70 genes and NIHL is necessary.

Recent animal study showed that noise exposure can induce cochlear neuronal degeneration, even when hearing thresholds return to normal [31]. This type of cochlear neural degeneration has been described as “hidden hearing loss”[32]. The noise-induced primary degeneration of cochlear nerve synapses did not affect hearing thresholds, but likely led to deficits in hearing abilities in difficult listening environment[33]. Subjects with hidden hearing loss may be more susceptible to noise, but there was no study that investigated the relationship between hidden hearing loss and susceptibility to NIHL. Thus, further research on the association between them is needed.

## Supporting information

S1 Data Supporting data. (SAV)

S1 Table Interaction between the SNPs and individual/environmental factors. (DOCX)
