# Supplementary material for: Polymorphisms of heat shock protein 70 genes (HSPA1A, HSPA1B and HSPA1L) and susceptibility of noise-induced hearing loss in a Chinese population: A case-control study
Source: PLoS One. 2017 Feb 9;12(2):e0171722. doi: 10.1371/journal.pone.0171722 (PMC5300111; doi:10.1371/journal.pone.0171722)
Supplement: S1 Table — (DOCX) [file pone.0171722.s002.docx]

**Supplement table Interaction between the SNPs and individual/environmental factors**

| SNPs | P_1_ | P_2_ | P_3_ | P_4_^*^ |
| --- | --- | --- | --- | --- |
| rs1043618 | 0.601 | **0.009** | 0.678 | 0.424 |
| rs2763979 | **0.029** | **0.001** | 0.100 | 0.339 |
| rs2075800 | 0.176 | 0.171 | 0.473 | 0.922 |
| rs2227956 | 0.660 | 0.242 | 0.912 | 0.786 |

* P_1_ to P_4_ represent P values for interactions between a SNP and CNE, smoking, drinking and hypertension status, respectively
